# Microbiota–Host Interactions: Exploring Their Dynamics and Contributions to Human Diseases

**DOI:** 10.1002/mbo3.70043

**Published:** 2025-08-06

**Authors:** Siau Wui Chin, Zheng Yao Low, Wei Qi Tan, Adzzie Shazleen Azman

**Affiliations:** ^1^ School of Science Monash University Malaysia Jalan Lagoon Selatan, Bandar Sunway Subang Jaya Selangor Malaysia

**Keywords:** antimicrobial resistance, atopic dermatitis, dysbiosis, host–pathogen interactions, Inflammatory bowel disease, tooth decay

## Abstract

Dysbiosis is the imbalance of bacterial composition, which would otherwise change the human host's metabolic activities and usual microbiota distribution. The outcomes would be as clear as day: losing beneficial bacteria in exchange for the overgrowth of potentially pathogenic bacteria, leading to diseases. It is crucial to unravel the dynamic roles of bacteria in maintaining human health to prevent and alleviate the said dysbiosis. To date, diet, lifestyle, age, and chemical exposures were cited as the leading cause of bacterial dysbiosis atop of genetic factors. This review aims to shed light on how bacterial interplays in maintaining human health and how bacteria–bacteria interaction may play a part in the surge of antimicrobial resistance. The intricate relationship of bacteria dynamics in the gut, skin and oral was detailed to understand how bacteria dysbiosis causes diseases such as irritable bowel syndrome (IBS), inflammatory bowel disease (IBD), acne vulgaris (AV), atopic dermatitis (AD), periodontitis and dental caries. Besides that, current interventions and limitations of therapeutic prospects entailing the growing concepts of rebiosis, including probiotics, prebiotics, synbiotics, microbiota transplantation, and the evolving phage therapy, were also discussed to breathe new life into the development of novel therapeutics against dysbiosis.

## Graphical abstract

Bacterial dysbiosis results from microbiota imbalances, diet, lifestyle, and other factors that lead to conditions such as IBS, IBD, acne, dermatitis, and dental diseases. The current therapeutic approaches include probiotics, prebiotics, synbiotics, microbiota transplantation, and phage therapy.

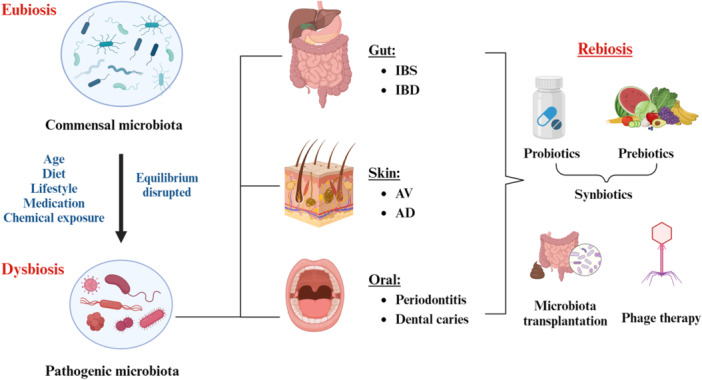

## 1 Introduction

Bacteria, a no‐stranger term to many, is an etiological agent contributing to various fatal diseases. The term “bacteria” has instilled fear in many, driven by the widely recognised severity of bacterial infections in many diseases. However, it is crucial to understand that humans and microbiomes are inseparable; humans are supra‐organisms that co‐evolve with the microbiomes colonising specific niches in the human body, living together symbiotically (Reynoso‐García et al. 2022). Bacteria have been commonly associated with being “bad”; for instance, *Porphyromonas gingivalis* and *Fusobacterium nucleatum* can cause oral squamous cell carcinoma (OSCC); *Staphylococcus epidermidis* and *Staphylococcus aureus* have been implicated in the development of atopic dermatitis, while an elevated presence of bacteria from the Enterobacteriaceae family is associated with irritable bowel syndrome (IBS) (Bjerre et al. 2021; Salem et al. 2018; Whitmore and Lamont 2014). However, there are numerous beneficial ones known as probiotics (e.g., the *Bifidobacteria* spp.) that help us to fend off harmful bacteria and, of course, entailing the ones that aid in various food manufacturing (e.g., the *Lactobacillus* spp.).

In the grand scheme of things, the elimination of pathogenic bacteria has become the focus to many, especially with the alarming increase in bacteria resistance towards numerous drugs, also known as antimicrobial resistance (AMR), which has further brought much‐needed awareness and highlighted the need for an effective antibacterial drug to eradicate pathogenic bacteria. According to the WHO, there has been a petrifying 42% third‐generation cephalosporin‐resistant *Escherichia coli* (*E. coli*) and 35% methicillin‐resistant *Staphylococcus aureus* across 76 countries as of 2022 (World Health Organization 2023). The Lancet review showed a staggering 4.95 million deaths associated with AMR in 204 countries, with *Escherichia coli*, *Staphylococcus aureus*, *Klebsiella pneumoniae*, *Streptococcus pneumoniae*, *Acinetobacter baumannii*, *Pseudomonas aeruginosa* being the leading cause, accounted for 3.57 million deaths in 2019 (Murray et al. 2022). In addition, the CDC reported a staggering 2.8 million AMR annual infections in the United States, with more than 35,000 fatalities in 2019, and it costs the Earth bills amounting to 4.6 billion US dollars to treat such infections (CDC Centers for Disease Control and Prevention 2024). The World Bank estimates that AMR will cost 1 trillion US dollars in additional costs by 2050 (World Health Organization 2023).

Henceforth, there is a call for studying the interaction between bacterial species to understand further how this interaction may be a therapeutic alternative for various diseases. It has been known that bacteria interact with each other in our body, either symbiotically or pathogenetically. For instance, the commensal bacteria found in the colon, including *Bacteroidetes*, *Firmicutes, Actinobacteria*, *Proteobacteria*, and *Verrucomicrobia*, work together to promote the growth of commensal bacteria at the outer mucus layer and foster a stable renewal of epithelial cells (Y. Chen et al. 2021). However, when this mucus is disrupted by mucus‐degrading bacteria such as the *Ruminococcus gnavus* and *Ruminococcus torques*, it will lead to the decline of *Bacteroides fragilis*, attracting pathogenic bacteria invasion and eventually contributing to inflammatory bowel disease (IBD) (Y. Chen et al. 2021). A similar imbalance in the microbiota can occur in both the skin and the oral cavity. On the skin, the transition of *Cutibacterium acnes* from a commensal to a pathogenic state can lead to conditions like acne vulgaris. In the oral cavity, a disruption involving *Streptococcus mutans* and other acidogenic bacteria contributes to the development of dental caries (Cavallo et al. 2022; E. Hajishengallis et al. 2017).

Dysbiosis is a condition where there is an imbalance in microbial equilibrium within a body region when the homeostasis is being disrupted (DeGruttola et al. 2016). This can mainly occur in three general ways, including the loss of commensal microbes, excessive growth of pathogenic microbes and the loss of overall microbial diversity (DeGruttola et al. 2016). Dysbiosis can lead to various diseases, ranging from gut and skin to oral diseases, involving bacteria and the host immune system interplay. Although there is a wide array of therapeutic options for the diseases caused by dysbiosis, little has been delved into the roles of bacteria in the diseases.

**Figure 1:**
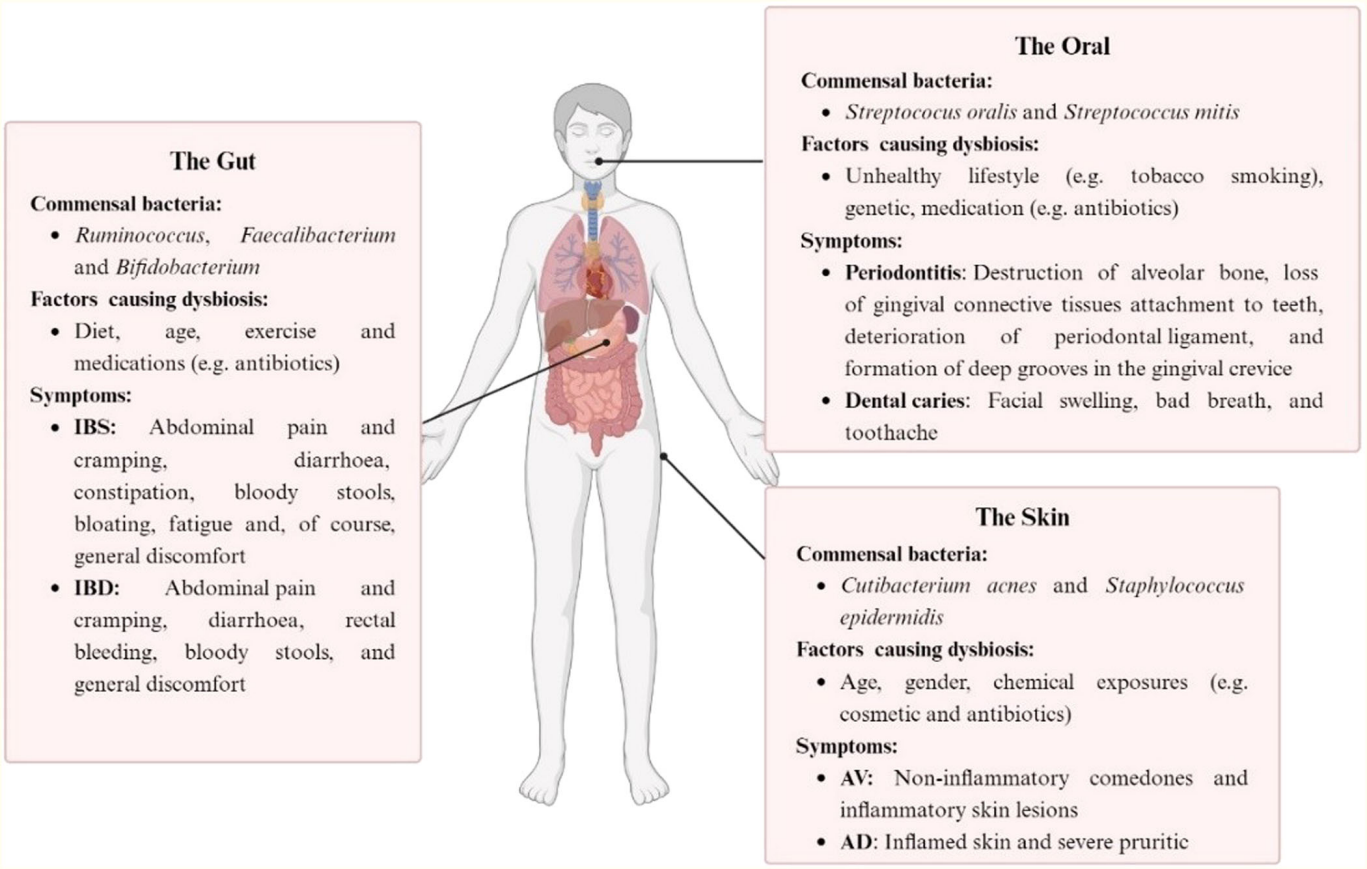
Summary of the commensal bacteria, factors affecting the microbiota and the symptoms of the diseases for each body part (the gut, skin, and oral). AD, atopic dermatitis; AV, acne vulgaris; IBD, inflammatory bowel disease; IBS, irritable bowel syndrome. The figure was created using BioRender.com.

In light of the above, this review aims to unravel and detail how the bacterial interactions between host–bacteria and bacteria–bacteria may shed new light to curb the perturbing rise of AMR and fatal bacterial infections. A few diseases in the gut, skin, and oral that are caused by bacterial dysbiosis will be discussed and summarized (Table 1 and Figure 1). In addition, some interventions highlighting the re‐establishment of a healthy complex microbiota (rebiosis), including the biotics (pre‐, pro‐ and synbiotics), microbiota transplantation, and phage therapy, will be briefly outlined, to pave the way for developing novel therapeutics against dysbiosis.

**Table 1:** Microbiota dysbiosis of different body parts (the gut, skin, and oral) and their respective diseases.

Disease	Microbiota alteration during dysbiosis	References
**The Gut**		
IBS	Increased: *Ruminococcus*, *Clostridium cluster XIVa* (Lachnospiraceae family, e.g., *Clostridium coccoides*), *Bacteroidetes*, *Lactobacillus*, *Veillonella*, and *Dorea* genera Enterobacteriaceae family Firmicutes/Bacillota and Proteobacteria phyla Decreased: *Bacteroidetes*, *Bifidobacteria*, *Faecalibacterium* spp. genera Actinobacteria phylum Coliforms	Rajilić–Stojanović et al. (2011), Salem et al. (2018)
IBD	Decrease in microbiota diversity at up to 25% Diminished Firmicutes phylum such as *Faecalibacterium prausnitzii* Overgrowth of *Enterobacteriaceae* such as *E. coli* Ileal CD: Increased *Enterobacteriaceae* and *Ruminococcus gnavus*; loss of *Faecalibacterium*	Rigottier‐Gois (2013), Willing et al. (2010)
**The Skin**		
AV	Increased: *Cutibacterium acnes* (predominantly), *Staphylococcus epidermidis*, *Staphylococcus aureus* Loss of: *Cutibacterium acnes* phylotypes diversity	Cavallo et al. (2022)
AD	Increased: *Staphylococcus aureus* (in severe AD), *Staphylococcus epidermidis* (in mild AD) Decreased: *Staphylococcus hominis*, *Cutibacterium acnes*	Bjerre et al. (2021), Byrd et al. (2017))
**The Oral**		
Periodontitis	Increased: Red complex—*Porphyromonas gingivalis, Treponema denticola*, and *Tannerella forsythia* Other contributors—*Bucteroides forytbus, Prevotella intermedia, Actinobacillus actinomycetemcomitans, Campylobacter rectus*, and *Fusobacterium nucleatum* Phyla Bacteroidetes and Firmicutes Decreased: Phyla Proteobacteria and Actinobacteria	G. Hajishengallis and Lamont (2012), Sedghi et al. (2021), Van Winkelhoff et al. (2002)
Dental caries	Increased: *Streptococcus mutans* (main etiological agent), *Streptococcus sobrinus*, *Lactobacilli*, *Bifidobacterium* spp., *Scardovia* spp., and *Actinomyces gerencseriae* Decreased: *Streptococcus sanguinis*	Aas et al. (2008), E. Hajishengallis et al. (2017), Valm (2019), B. Zhu et al. (2018), Y. Zhu et al. (2023)

Abbreviations: AD, atopic dermatitis; AV, acne vulgaris; IBD, inflammatory bowel disease; IBS, irritable bowel syndrome.

## 2 The Gut

Gut bacteria have the most significant and convoluted functions and roles in the human body. The recorded estimate of human gut microbiota is at a staggering 1000 species‐level operational taxonomic units (OTUs) in humans (Shapira 2016). Depending on the pH, oxygen tension, host secretions, and digestive activities in the gut, there are different dominant microbial colonisers (Flint et al. 2012). Of these, the Bacteroidetes, Firmicutes/Bacillota, Proteobacteria and Actinobacteria are the four dominant phyla, with the former two being greater in abundance (90%) and mainly residing in the jejunal and ileal (Binda et al. 2018; Flint et al. 2012; Ramakrishna 2013). Concerning the overall density of bacterial genera in the gut, the *Bacteroides*, *Clostridium*, *Peptococcus*, *Bifidobacterium*, *Eubacterium*, *Ruminococcus*, *Faecalibacterium*, and *Peptostreptococcus* remain to be the most recorded ones (Gomaa 2020).

The balance of these bacteria is of notable significance, especially concerning the derivation of nutrition and synthesizing the essential vitamins that are non‐readily produced (e.g., vitamin B12). Studies have shown that between 10% and 20% of starch ingested every day is resistant to amylase digestion (Resistant Starch; RS), which includes the non‐starch polysaccharides (NSPs), with food processing adding to a greater percentage (Ramakrishna 2013). To be absorbed by the colon, the RS has to be converted to short‐chain fatty acids (SCFAs) such as acetate (C2), propionate (C3) and butyrate (C4) at a relative proportion of 60:20:20, and these have to be fermented by the abovementioned colonic bacteria (Portincasa et al. 2022). As such, *Ruminococcus* and *Faecalibacterium* ferment carbohydrates into butyrate and *Bifidobacterium* for hydrolysation of starch and produce acetate, to name a few. SCFA has been well known to modulate several metabolic pathways, which involve obesity, insulin resistance, and type 2 diabetes (Portincasa et al. 2022). Besides that, the biosynthesis of essential vitamin B complex has also been reported involving enterotype I (*Bacteroides* spp.) and enterotype 2 (*Prevotella* spp.) (Arumugam et al. 2011). The gut microbiome also plays a significant role in calcium absorption and bone development. Studies have pointed towards the reduced pH environment from the SCFA production, butyrate, particularly by the abovementioned bacteria, inducing calcium retention in bones. The effect mentioned was apparent in the study conducted by Weaver (2015), where increased calcium and magnesium absorption and retention were accompanied by increased femur and tibia bone density with increased galacto‐oligosaccharides (GOS) administration for enhanced bifidobacteria proportion in male mice model (Weaver 2015). The same study has also predicted a 12% higher fractional calcium absorption over 1‐year of prebiotic consumption in humans, which would have accounted for 1.8% of whole‐body bone mineral content (BMC) (Weaver 2015).

The intricacies between the microbiome, host metabolism, and immune system extend to mental and neurological functions. As reported by Ullah and colleagues, there has been increasing evidence of the possibility of gut dysbiosis for neurological disorders, including the widely seen Alzheimer's disease, Parkinson's disease, multiple sclerosis and autism spectrum disorder (ASD) via the communication of gut–brain axis (GBA) (Cryan et al. 2020; Ullah et al. 2023). The perplexing of the abovementioned continues to develop with recent evidence showing the involvement of the SCFA gut bacteria in the maturation of microglia, the innate immune cells in the brain. On top of that, the gut microbiome has the capacity to produce neurotransmitters that each have a specific impact on the brain γ‐aminobutyric acid (GABA), an amino acid that functions to inhibit the neurotransmitters to the central nervous system (CNS) (J. Chen et al. 2016). For instance, *Lactobacillus* and *Bifidobacterium* can produce GABA and modulate different neurological parameters, including sleep, appetite, mood, cognition and psychiatric (Duranti et al. 2020).

### 2.1 Factors Affecting Gut Microbiota

Undoubtedly, the entanglement of the tightly regulated gut microbiome in metabolism, immune, mental and neurological functions can also be susceptible to dysregulation and dysfunction. This was driven by various factors, ranging from diet, age, exercise, genetics, smoking, and antibiotics, to name a few.

Depending on the dietary preference, the gut microbiome may differ vastly. For instance, infants consuming rich oligosaccharide breast milk resulted in the increased expression of immunoglobulin G (IgG) due to increased SCFA production via enhanced *Actinobacteria* (particularly the genus *Bifidobacterium*) growth and suppression of Firmicutes and Proteobacteria (Gomaa 2020). Formula‐fed infants, however, exhibited different gut microbiome profiles with lower *Actinobacteria* and higher Bacteroidetes and Firmicutes phyla than breastfed infants (Thompson et al. 2015). A diet with saturated fats, mainly animal sources, lowers beneficial *Bifidobacterium*, Bacteroidetes, *Prevotella*, and *Lactobacillus* and promotes inflammation (Ramos and Martín 2021). A diet with monounsaturated, medium‐chain, and n‐3 polyunsaturated fatty acids, on the other hand, was reported to increase the beneficial Bacteroidetes, increasing SCFA production, thereby preventing obesity and its related metabolic disease (Machate et al. 2020).

Age is also one of the factors affecting the gut microbiome. A healthy adult would have predominant Firmicutes and Bacteroidetes in the gut as opposed to the adolescent, with significantly higher *Clostridium* and *Bifidobacterium* (Biagi et al. 2017). While dietary, as mentioned earlier, plays a significant role in the gut microbiome, the development of different dietary habits (e.g., rich in saturated fats), reduced physical activity, and declining health status of older adults with the use of multiple drugs can all contribute to inflammageing that would expedite the decrease of SCFAs producing bacteria, for example, Firmicutes and Bacteroidetes. This would also give way to opportunistic pathogens such as *streptococci*, *staphylococci*, *Enterobacteria*, and *Enterococci* in older adults (Biagi et al. 2017).

Exercise has been postulated to drive one's physical and mental health. In the human gut microflora, exercise has shown a greater abundance of Firmicutes phyla and SCFAs production genera, for example, *Ruminococcaceae*, than non‐athletes (Clarke et al. 2014).

Antibiotics, a commonly prescribed medication for various forms of infections, typically include beta‐lactam antibiotics, aminoglycosides, daptomycin and linezolid, to name a few (Dahiya and Nigam 2023). These antibiotics disturb the gut microflora. For instance, a study has shown that Clarithromycin, a macrolide antibiotic used to treat pneumonia, skin infections, strep throat,t and many more, decreases the Enterobacteria phyla, *Bifidobacterium* sp., and *Lactobacillus* sp. for up to 5 weeks in the gut (Elvers et al. 2020).

### 2.2 Notable Diseases From Gut Dysbiosis

#### 2.2.1 Irritable Bowel Syndrome (IBS)

IBS is part of a chronic functional gastrointestinal disorder (FGID), primarily affecting the stomach and intestines. It is characterized by a combination of symptoms, including abdominal pain and cramping, altered bowel habits (diarrhoea, constipation, or both), bloating, fatigue, and general gastrointestinal (GI) discomfort (Sood et al. 2014). IBS affects 5%–10% of the global population and is often believed to have a higher association with females, although it remained fully unravelled, affecting the quality of life of many (Black 2021; Pimentel and Lembo 2020).

To date, there is no gold standard diagnosis or biomarkers for IBS. Thus, researchers have constantly attempted to develop simplified diagnosis criteria for IBS, such as Manning, Kruis, and Rome (B. Lacy and Patel 2017). Of these, Rome criteria continue to grow and evolve to account for the complication of IBS diagnosis where IBS generally can be diagnosed and further subdivided into four subtypes as depicted by the Rome IV classification, namely the IBS‐D (diarrhoea predominant), IBS‐C (constipation‐predominant), IBS‐M (mixed diarrhoea and constipation), and IBS‐U (nonclassified) (B. Lacy and Patel 2017). Rome IV, the latest revision, defined IBS as a functional bowel disorder where abdominal pain during defaecation and change of bowel habits such as constipation, diarrhoea or a mix of both with symptoms onset at least 6 months before diagnosis and symptoms should present during the last 3 months of onset (B. E. Lacy et al. 2016).

It is understood that the intricacies of human gut microbiota are pronounced at a staggering 1000 species‐level operational taxonomic units (OTUs) that have different dominance depending on the pH, oxygen tension, secretions, and many more (Shapira 2016). Although the exact cause of IBS has yet to be fully unravelled, it was postulated that it has to do with gut‐brain interaction and thus can also be classified as a neuro‐gastrointestinal (GI) disorder such as visceral hypersensitivity and dysmotility, where the former may have greater activation of the amygdala for enhanced pain and perceptive responses in the GI tract that is super sensitive to pain and the latter, have more contractions on the colon causing cramps (Bokic et al. 2015; Mayer et al. 2005). Apart from that, there is also evidence pointing towards stress, where it could activate the hypothalamic‐pituitary axis that mediates low‐level inflammation and mast cell infiltration that leads to heightened mediators' production, such as the serotonin that contributes to the sensational pain and in the bowel (Philpott et al. 2011).

The major potential cause for IBS by no means is gut dysbiosis, as depicted in the earlier section, including but not limited to diet, age, exercise, and use of antibiotics. Of these, food was postulated to be the leading cause of IBS. It is noted that 10%–20% of starch ingested every day is resistant to amylase digestion and has to be converted to SCFA by colonic bacteria such as *Ruminococcus*, *Faecalibacterium* and *Bifidobacterium* (Ramakrishna 2013). The primary product of Fermentable oligosaccharides, disaccharides, monosaccharides, and polyols (FODMAPs) are SCFAs, which may heighten the prevalence of the mentioned fermentable colonic bacteria, such as *Ruminococcus* in IBS, triggering a slew of pathophysiological reactions such as abdominal pain and bloating as a result of gas production (Rajilić‐Stojanović et al. 2015). Thus, low‐FODMAP food was suggested for IBS, albeit lacking extensive composition, functionality and paradoxical reports concerning the effectivity and potential health benefit, especially concerning the product of SCFAs, butyrate, which was documented to be an essential source for the inhibition of inflammation and carcinogenesis in addition to reinforcement of colonic defence barrier and reduction of oxidative stress (Hamer et al. 2008).

IBS can be generally characterized by an increase in a slew of bacterial genera, including *Ruminococcus*, *Clostridium cluster XIVa* (Lachnospiraceae family, e.g., *Clostridium coccoides*), *Bacteroidetes*, *Lactobacillus*, *Veillonella*, and *Dorea*. In addition, the Enterobacteriaceae family, Firmicutes/Bacillota and Proteobacteria phylum are also found to be increased in IBS (Salem et al. 2018). This was followed by a significant decrease in bacteria genera such as the *Bacteroidetes*, *Bifidobacteria*, *Faecalibacterium* spp. and Actinobacteria phylum and coliforms (Rajilić–Stojanović et al. 2011; Salem et al. 2018). In terms of the four subtypes of the Rome IV classification, a study by Qi and colleagues showed that IBS‐D and IBS‐U have a decrease in Firmicutes, *Actinobacteriota*, *Verrucomicrobiota*, and Campilobacterota and an increase in Proteobacteria. Conversely, IBS‐C showed a rise in *Verrucomicrobiota* and *Desulfobacterota* (Su et al. 2023).

#### 2.2.2 Inflammatory Bowel Disease (IBD)

IBDs are long‐term chronic inflammation of the digestive tract that can be characterized into two different forms: ulcerative colitis (UC) and Crohn's disease (CD) (Irving and Gibson 2008). As the name suggests, UC forms ulcer sores along the superficial lining of the colon and rectum, while CD involves deeper layers of the entire GI tract that primarily focus on the small intestine and cause multiple complications, such as fistulas and abscess formation, to name a few (Van Der Sloot et al. 2017). Akin to IBS, IBD affects the stomach and intestines; symptoms often include abdominal pain and cramping, diarrhoea, rectal bleeding, bloody stools, and general discomfort, leading to delayed diagnosis (Yu and Rodriguez 2017). IBD affects 59.25 per 100,000 people globally, with greater prevalence rates at ages 50–54 in females and 60–64 years in males (R. Wang et al. 2023). Albeit the greater prevalence in females, the cause of IBD has yet to be fully unravelled and is believed to be highly associated with genetic factors.

Diagnosing IBD can be challenging given the intermittent occurrence and similar symptoms to IBS and other more common conditions. The lack of a suitable marker, in addition to the onset of IBD, often begins with extra‐intestinal manifestations (up to 43% of patients with CD) such as arthritis, psoriasis, erythema nodosum, primary sclerosing cholangitis, to name a few, prompting high chances of delayed or misdiagnosis (Card et al. 2014; Roda et al. 2020). Akin to IBS, there is no single perfect diagnosis or biomarkers for IBD, and thus, several tools have been developed to assess the severity of CD, such as the Crohn's Disease Activity Index (CDAI) and the Crohn Disease Endoscopic Index of Severity (CDEIS) where the former accounts for eight factors such as the number of liquid or soft stools daily—7 days, abdominal pain (severity ranges from 0 to 3) daily—7 days, general well‐being (severity ranges from 0 to 4) daily—7 days, extra‐intestinal manifestations, opiates for diarrhoea, abdominal mass (ranges from 0 to 5), haematocrit value (men < 0.47, women < 0.42) and percentage deviation body weight from standard (Khanna et al. 2015). In contrast, CDEIS look into four different parameters such as deep ulcerations, superficial ulcerations, surface involved by disease, and surface involved by ulcerations, with scores < 3 indicating remission, mild activity (3–9), moderate activity (9–12), and severe activity (≥ 12) (Karczewski et al. 2015).

Akin to IBS, the exact cause of IBD has yet to be fully understood. Many studies have pointed towards different lifestyles, environmental, diet, and genetic factors. Higher levels of obesity contributed by the westernization of diet, that is characterized by high levels of saturated fat, red and processed meat, has also been partially attributed to IBD. This is evident in the increased growth of *Bilophila wadsworthia* from an animal‐based diet, damaging intestinal tissues and subsequently triggering IBD (Devkota et al. 2012). Rural subjects have significantly less bacterial diversity and Bacteroidetes population than urban subjects, possibly a factor for the development of IBD (Rosas‐Plaza et al. 2022). Genetics is by no means a common influence on many diseases. The multidrug resistance 1 (MDR1) gene that governs the efflux of drugs in humans is associated with UC and CD; as such, the MDR1‐deficient mice model showed colitis (Sartor 2006). The use of antibiotics, as mentioned earlier, will also trigger different degrees of inflammation depending on the dosage, frequency of use, and drug types. Typically, nonsteroidal anti‐inflammatory drugs (NSAIDs) are highly correlated with CD (Chan et al. 2011; Gecse and Vermeire 2018). These factors make the conventional IBD treatments challenging, which include the use of aminosalicylates, corticosteroids, immunomodulators, and surgical intervention in cases of complications, primarily to alleviate inflammatory symptoms and achieve sustained remission (Cai et al. 2021; Saeid Seyedian et al. 2019).

Despite there being limited conclusive clinical studies on dysbiosis as a factor of IBD, a cohort study by Willing and colleagues demonstrated that patients with ileal CD showed increased levels of *Enterobacteriaceae* and *Ruminococcus gnavus*, followed by the disappearance of core microbiota such as *Faecalibacterium* (Rigottier‐Gois 2013; Willing et al. 2010). IBD can generally be characterized by a decrease in microbiota diversity at up to 25% and diminished Firmicutes phylum in healthy gut microbiota, such as the *Faecalibacterium prausnitzii* (Rigottier‐Gois 2013). Concurrently, the overgrowth of *Enterobacteriaceae*, such as *E. coli*, was also observed (Rigottier‐Gois 2013).

## 3 The Skin

The skin, being the largest organ in the human body, is the first line of defence against harmful pathogens. This is attributed to the microbial community, which synergistically regulates the balance of the skin microbiota in addition to the physical barrier to prevent the colonization of pathogens (Byrd et al. 2018). The skin microbiota has an average microbial density of 10^3^ to 10^6^ CFU/cm^2^ with over 200 characterized genera, the second‐greatest microbial density after the gut (Smythe and Wilkinson 2023). Of these, the four dominant phyla are the Actinobacteria, Firmicutes, Proteobacteria and Bacteroidetes, and the noted common genera are the *Staphylococcus*, *Propionibacterium*, *Corynebacterium*, and *Streptococcus* (Reynoso‐García et al. 2022). The microbial composition varies depending on the skin sites (oily, moist, and dry regions). For instance, the *Propionibacterium* species, particularly *Propionibacterium acnes* (latter known as *Cutibacterium acnes*), were found dominantly at the anoxic sebaceous skin sites due to its facultative anaerobic properties (Reynoso‐García et al. 2022). Meanwhile, humid skin sites predominantly consist of *Staphylococcus* and *Corynebacterium* spp. (Reynoso‐García et al. 2022).

In a homeostatic state, an immunity barrier is created by the symbiotic relationship of skin microbiome and immune cells to prevent the invasion of pathogenic microbes. *Cutibacterium acnes* (*C. acnes*) plays a key role in maintaining this barrier by producing lipases, which break down lipids in sebum to release free fatty acids, primarily propionic acid, establishing an acidic skin environment that discourages the colonization of harmful pathogens (Flowers and Grice 2020). The acidic condition inhibits the growth of common pathogens, including *Staphylococcus aureus* (*S. aureus*) and *Streptococcus pyogenes* (*S. pyogenes*). Still, coagulase‐negative staphylococci (CoNS) and Corynebacteria growth are favoured (Grice and Segre 2011). The *Staphylococcus epidermidis* (*S. epidermidis*), another dominant innocuous species, has been found to inhibit the colonization of pathogenic bacteria on the skin by producing the alpha‐helical peptides, phenol‐soluble modulins (PSMs) (Sabaté Brescó et al. 2017). PSMs share structural and functional similarities with host‐derived antimicrobial peptides (AMP) that target bacterial cell membranes, except *S. epidermidis*, causing cell content leakage (Sabaté Brescó et al. 2017). In sum, the *S. epidermidis* δ‐toxin (PSMs γ) and host‐derived AMPs were proven to exhibit synergistic antibacterial activities (Cogen et al. 2010).

Besides that, *S. epidermidis* produces lipoteichoic acid, which reduces skin inflammation through toll‐like receptor (TLR) 2 signalling activation (Lai et al. 2010). Notably, *S. epidermidis* protects epidermal keratinocytes by inducing the production of human beta‐defensins (hBD) 2 and hBD3 that penetrate bacterial cell membranes (Lai et al. 2010). Furthermore, *S. epidermidis* showed a reduction of viable group A *Streptococcus* (GAS) at the local infection site of the mice treated with *S. epidermidis*, conferring protection against GAS skin infection (Lai et al. 2010). On the other hand, *S. epidermidis* that secretes serine protease Esp has been shown to outcompete and induce sessile to planktonic change, an apparent degradation of biofilms in pathogenic bacteria such as *S. aureus*, methicillin‐resistance *S. aureus* (MRSA) and vancomycin‐intermediate *S. aureus* (VISA) (Iwase et al. 2010). Moreover, Esp enhances the bactericidal effect of hBD2 against *S. aureus* in biofilms (Iwase et al. 2010). Interestingly, *S. epidermidis* exerts inhibitory effects against *C. acnes* by fermenting glycerol into short‐chain fatty acids that inhibit *C. acnes*, regulating *C. acnes* abundance and maintaining skin homeostasis (Sabaté Brescó et al. 2017). These skin commensals, however, can shift into opportunistic pathogens when dysbiosis occurs, causing skin diseases such as acne vulgaris (AV) and atopic dermatitis (AD).

### 3.1 Factors Affecting Skin Microbiota

The abundance, composition, and distribution of skin microbes vary by intrinsic (age, genetic, and immunity) and extrinsic factors (hygiene, physical activity, and chemical exposure) (Skowron et al. 2021). The difference in skin microbiota is apparent among individuals of different ages. Although birth mode could be a factor, it only lasts for the first hour to days of life. The infants' skin microbiota is generally Firmicutes rich, including staphylococci and streptococci (Dhariwala and Scharschmidt 2021). The Firmicutes' abundance reduces over age, with the greater significance of Actinobacteria, especially the *Cutibacterium acnes* species, as puberty hits due to increased skin sebum secretion and hormonal alterations (Oh et al. 2012). The shift doesn't stop here; as we age, the increased Proteobacteria supersede the Actinobacteria due to natural changes such as reduced sebum secretion and immunity (Jugé et al. 2018).

Generally, males produce more steroids than females, thus conferring greater growth to *Staphylococcus*, *Propionibacterium*, and *Corynebacterium* that utilize sebum as nutrients (Fierer et al. 2008; Kim et al. 2021). *S. aureus*, specifically, was found abundantly in males, which can be associated with lower skin moisture levels and a more significant transepidermal water loss (Kim et al. 2021). Conversely, Enterobacteriales and Lactobacillaceae were more predominant in females, possibly attributed to better hygienic behavior, such as greater hand wash frequency (Fierer et al. 2008).

Chemical exposures, such as the use of cosmetics and antibiotics, can also alter skin microbiota. For instance, *Staphylococcus* and *Propionibacterium* were found to be more abundant in individuals who use moisturisers due to their lipid compositions (Bouslimani et al. 2019). Minocycline, an antibiotic commonly used to treat skin diseases such as acne, has been found to reduce the level of *Cutibacterium* (predominant acne‐causing bacteria) and *Lactobacillus* (beneficial bacteria) and elevate the level of *Pseudomonas* (common skin infection bacteria) and *Streptococcu*s (common upper respiratory tract infection bacteria), a potential leading cause to skin dysbiosis complications including inflammation, irritation and itchy skin (Chien et al. 2019; Skowron et al. 2021). Consistent or inappropriate use of antibiotics may lead to the development of antibiotic resistance in skin bacteria (Skowron et al. 2021).

### 3.2 Notable Diseases From Skin Dysbiosis

#### 3.2.1 Acne Vulgaris (AV)

AV is a chronic inflammatory skin disease affecting approximately 9.38% of people globally and 85% of teenagers (Mohsin et al. 2022). AV is primarily characterized by increased sebum secretion (hyperseborrhea), resulting in noninflammatory comedones and inflammatory lesions. Other key factors contributing to AV include altered follicular keratinization, inflammation, and colonization of pathogenic *C. acnes*. These pathophysiologies are interconnected, with exposome factors such as hormonal changes and genetic predisposition contributing to hyperseborrhea and hyperkeratinization. Together, these factors lead to keratotic plug formation that occludes the pilosebaceous ducts and promotes the development of comedones (Cunliffe et al. 2000). The persistent sebum accumulation and duct occlusion will lead to a hypoxic condition of the folliculopilosebaceous unit and favour the inhabitation of facultative anaerobic *C. acnes*. The standard treatments for AV include topical retinoid therapy, antibiotics (such as clindamycin and erythromycin), and oral isotretinoin, depending on the severity (Mohiuddin 2019; Vasam et al. 2023). However, these treatments have downsides, such as teratogenicity, dermatologic and ocular reactions and antibiotic resistance, allowing active research on their alternative.

Notably, in the event of acne, *C. acnes* shifts from a “friend” to a “foe,” causing dysbiosis of the skin barrier and inducing inflammation. As dysbiosis occurs, the proliferation of acneic *C. acnes* increases, leading to the loss of *C. acnes* phylotype diversity without altering their relative abundance. *C. acnes* was stratified into phylogenetic clades IA1, IA2, IB1, IB2, IB3, IC, II, and III, which were further classified into ribotypes (RTs) to distinguish the healthy and acneic *C. acnes* strains (Fitz‐Gibbon et al. 2013). Phylotype IA1 (including RT 4, 5, and 8) is the predominant phylotype in acneic skin, while phylotypes IA2, IB or II are commonly associated with healthy skin (Fitz‐Gibbon et al. 2013). Dysbiotic shift within a follicle can also be associated with the biofilm formation by virulent *C. acnes*, particularly the phylotype IA1, and *S. epidermidis* (Cavallo et al. 2022). Skin bacterial biofilm is developed as the bacteria adhere tightly to the human skin and are embedded within a polysaccharides‐rich matrix of extracellular polymeric substance (EPS) (Fournière et al. 2020). EPS acts as a barrier that protects bacteria and resists or slows down the antimicrobial agents' inflow, increasing the difficulty in treating acne. Biofilm formation tends to attract the colonization of other pathogenic bacteria, such as the common *S. aureus*, upregulating the secretion of virulence factors and aggravating acne development. Hence, dysbiosis in the skin can be characterized as the increased proliferation of virulent *C. acnes*, *S. epidermidis*, and *S. aureus*, accompanied by the loss of *C. acne* phylotypes diversity, eventually disrupting the balance of normal skin microbiota.

Dysbiosis of skin microbiota eventually leads to inflammation. Thus, the virulent *C. acnes* is often associated with inflammatory acne such as papules, pustules, nodules, and cysts. Acne‐related *C. acnes* strains increase and activate the secretion of virulence factors such as lipases, proteases, hyaluronate lyase, porphyrin, and Christine Atkins Munch‐Petersen (CAMP) factors, a primary contributor to skin inflammation (Cavallo et al. 2022). Lipases attract inflammatory cells, including neutrophils, to hydrolyse sebum into free fatty acids, leading to inflammation and hyperkeratosis. Proteases, hyaluronate lyase, and CAMP degrade the extracellular matrix (ECM) constituents, allowing the invasion of virulent *C. acnes* (Lee et al. 2019). The haemolytic activity of CAMP factors of *C. acnes* can be enhanced by *S. aureus* sphingomyelinase (SMase) by first hydrolysing the sphingomyelin on erythrocyte membranes, increasing the pore‐forming capability of CAMP (Nakatsuji et al. 2011). The increased porphyrin secretion by *C. acnes* will interact with keratinocytes, leading to potassium ion leakage and inducing inflammation (Spittaels et al. 2021). Porphyrins were also found to cause *S. aureus* aggregation and biofilm formation, indicating its role in interspecies interactions (Wollenberg et al. 2014).

The inflammatory responses induced by *C. acnes* can be subjected to sebocytes, keratinocytes, and peripheral blood mononuclear cells (Lee et al. 2019). The activation of TLR‐2 and TLR‐4 by *C. acnes* on keratinocytes and sebocytes induces the nuclear factor (NF)‐κB pathways that release pro‐inflammatory chemokines such as interleukin (IL)‐1, IL‐6, IL‐8, tumour necrosis factor‐α (TNF‐α), and hBD2 (Lee et al. 2019). Upon recognizing *C. acnes*, macrophage/monocyte antigen CD‐36 in keratinocytes produces reactive oxygen species (ROS) that heighten the innate immune response to eradicate the pathogenic *C. acnes*, causing inflammation (Lee et al. 2019). Concurrently, adaptive immunity induced by *C. acnes* via the differentiation of naïve CD4^+^ CD45RA T lymphocytes into T helper (Th) 1 and Th17 cells increases the secretion of IL‐17 and interferon (IFN)‐γ, further enhances inflammation (Mouser et al. 2003).

#### 3.2.2 Atopic Dermatitis (AD)

AD is a chronic, non‐communicable and recurring inflammatory skin condition that has a tremendous impact on skin diseases, primarily affecting children (20%) compared to adults (up to 10%) (Laughter et al. 2021). Being the most common type of eczema, AD is characterized by inflamed skin and severe pruritus that can affect nearly the entire skin surface. The multifaceted pathogenesis of AD involves the interplay between genetic and environmental factors, which results in skin barrier dysfunction and immune system dysregulation (Byrd et al. 2017). Topical corticosteroids are the first‐line treatment for mild to moderate AD, whereas topical calcineurin inhibitors (TCIs) serve as steroid‐sparing alternatives for the treatment of moderate to severe AD, both exerting anti‐inflammatory effects (Afshari et al. 2024; Bhatt et al. 2024). Despite their efficacy, these therapies are associated with significant adverse effects such as harbouring risks of skin atrophy, striae, burning and stinging sensations, thus are generally unsuitable for prolonged usage (Afshari et al. 2024; Bhatt et al. 2024).

AD is marked by the increased colonization of *S. epidermidis* and *S. aureus* in mild and severe AD, respectively (Byrd et al. 2017). The decreased bacterial diversity in AD is also apparent, where an increased colonization of *S. aureus* has resulted in a lower abundance of *Staphylococcus hominis* and *C. acnes* (Bjerre et al. 2021). Notably, noninflammatory responses were recorded on murine models applied with *S. epidermidis* extracted from AD patients. Conversely, the same setting showed epidermal thickening, inflammatory responses, and cutaneous infiltration of immune cells (Th2 and Th17 cells) on murine applied with *S. aureus*, indicating *S. aureus* being the main contributor to inflammation in AD (Byrd et al. 2017).

Apart from that, dysbiosis in AD was also attributed to the presence of PSMs in multi‐species biofilm formation by *S. aureus* and *S. epidermidis*, with the former showing greater biofilm propensity with increased AD severity, a predominant pathogenic contributor in AD (H. Chen et al. 2022; Gonzalez et al. 2021). The severed skin barrier, with reduced AMPs and free fatty acids production in AD patients, has led to an increased alkaline environment that favours the colonization of *S. aureus* (Paller et al. 2019). Generally, *S. aureus* exerts its pathogenicity via its cell wall proteins and secreted factors, which adhere to and destroy the skin barrier and facilitate pro‐inflammatory mechanisms. For instance, clumping factors (Clf) A and B and fibronectin‐binding protein (fnBP) of *S. aureus* help in adhering to the stratum corneum; *S. aureus* α‐toxin and proteases disintegrate the skin barrier; protein A and staphylococcal enterotoxin (SE) superantigens (SEA, SEB, SEC) induce B cell expansion and increase cytokine release to induce inflammatory reactions (Paller et al. 2019).

The secretions of *S. aureus* have led to various virulence mechanisms in AD. *S. aureus* stimulates Langerhans cells and T‐cell proliferation, which leads to the imbalanced Th2‐shifted immune responses and the production of IL‐1α via TLR9 activation (Iwamoto et al. 2019). *S. aureus* ClfA and α‐toxin induce Th1 cytokine secretion and activate Th1 to secrete IFN‐γ, respectively (H. Chen et al. 2022). PSMα is expressed by *S. aureus* to induce the cytolytic effect on neutrophils, and the secretion of IL‐1α, IL‐36α, and IL‐17 enhances inflammation (H. Chen et al. 2022). Proteases such as serine protease‐like proteins (Spls) of *S. aureus* trigger IgE antibody reactions in B cells, leading to atopic march in AD (H. Chen et al. 2022). Protein A can be internalised into keratinocytes along with *S. aureus*, which causes local skin inflammation via its binding to tumour necrosis factor receptor‐1 (TNFR‐1), contributing to the increased activation of NF‐κB pathways (Iwamoto et al. 2019). Meanwhile, SEB induces IL‐21 expression, leading to Th17 differentiation, which increases the secretion of pro‐inflammatory cytokines, chemokines, and other inflammatory mediators (Bauquet et al. 2009).

## 4 The Oral

Akin to the skin, the oral microbiome has also been recognised as the second largest in microbial diversity, consisting of more than 700 bacterial species, with the main phyla being Firmicutes, Actinobacteria, Proteobacteria, and Fusobacteria. Distinct mouth regions harbour different preponderant bacterial species, such as *Streptococcus mitis* (*S. mitis*) in buccal mucosa and *Streptococcus oralis* (*S. oralis*) in dental plaque (McLean et al. 2022). The diverse and complex oral microbial ecosystem can be attributed to the various microbial habitats (including teeth, tongue, and buccal mucosa) and nutritional uptake in each individual.

In a physiological state, the homeostasis of the oral cavity is maintained by the bacterial symbionts and the host's immune system to prevent oral infections. The symbiotic communication between oral microbiota and the host is established via TLR, where TLR‐2 and TLR‐4 are expressed by dendritic cells (DCs) to induce tolerance against the dense bacterial community in the oral cavity (Zaura et al. 2014). Neutrophils are essential in defending the gingival tissues, and the oral resident bacteria modulate their recruitment. The commensal oral bacteria regulate low expression levels of E‐selectin, intracellular adhesion molecule 1 (ICAM‐1), and the chemokines CXCL8 (also known as IL‐8), facilitating neutrophil extravasation and aggregation in the gingival tissues (Devine et al. 2015; Dixon et al. 2004). Despite being the primary source of oral pathologies upon its loss of diversity (with a single or few species predominating), the biofilm also maintains the balanced microbial community in the oral cavity. In addition, the secretory immunoglobulin A (S‐IgA) secreted by saliva and gingival crevicular fluid (GCF) helps regulate microbial adhesion and colonization (Zaura et al. 2014). In a healthy setting, the IgA proteases secreted by the commensal oral Streptococci, such as *S. oralis* and *S. mitis*, evade S‐IgA for survival (Zaura et al. 2014). However, the production of pathogenic IgA proteases was observed to neutralise S‐IgA, favouring the colonization of pathogens, leading to many pathological conditions. Although the abovementioned mechanisms play crucial roles in maintaining oral health, they also cause dysbiosis via the altered expression patterns of TLRs, altered balance in neutrophils transit, altered biofilm diversity, and the induction of pathogenic bacteria into the oral cavity, leading to oral pathologies.

### 4.1 Factors Affecting Oral Microbiota

Similarly, environmental, lifestyle, and genetic factors can affect oral microbiota, where altering the microbial composition, structure, and metabolic functions would increase individual susceptibility to developing oral diseases. Firstly, tobacco smoking is a well‐established risk factor for the alterations of the host immune response and oral microbiome. Smoking inhibits bacterial‐stimulated expression of superoxide and surface toll‐like receptor 2 (TLR2), thus impairing the phagocytotic activity of macrophages and inflammatory signalling pathways (Petersen and Round 2014). In addition, smokers have a greater abundance of Firmicutes, Bacteroidetes, and Actinobacteria and reduced Bacteroidetes, Fusobacteria, Proteobacteria, Neisseria, Branhamella, Porphyromonas, and Gemella phyla compared to non‐smokers (Darby 2022).

Genetic factors play a vital role in the progression of oral disease with consequent tissue destruction. Numerous studies have pointed towards genetic polymorphisms in the interleukins (IL‐1A, IL‐1B, IL‐6, IL‐10) and inflammatory mediators (MMP‐3, MMP‐9) genes to a strong association with the severity of adult periodontitis (Da Silva et al. 2017). Additionally, genetic predisposition factors can affect the quality and structure of enamel, where low‐birth‐weight children are more prone to enamel hypoplasia than normal birth‐weight children (Franco et al. 2007).

Undoubtedly, antibiotics greatly influence the metabolic functions and compositions of oral microbiota. The utilization of amoxicillin has been shown to increase antibiotic resistance and decrease the abundance of *Neisseria, Streptococcus*, and *Veillonella* in the oral microbiota (Kajiya and Kurihara 2021).

### 4.2 Notable Diseases From Oral Dysbiosis

#### 4.2.1 Periodontitis

Periodontitis is a chronic inflammatory periodontal disease in the oral cavity characterized by the destruction of alveolar bone, loss of gingival connective tissues attachment to teeth, deterioration of periodontal ligament, and formation of deep grooves in the gingival crevice (Suárez et al. 2020). This destructive process can ultimately lead to tooth loss if no medical attention is given. Affecting 5% to 30% of the adult population globally, primarily aged 25 to 75 years, severe periodontitis has also been widely associated with several inflammation‐driven systemic disorders, such as cardio‐metabolic, neurodegenerative, autoimmune diseases, and cancer (G. Hajishengallis and Chavakis 2021; G. P. Wang 2015). The symptoms of periodontitis include swollen or bleeding gum, persistent bad breath, and loose teeth. Periodontitis management includes both nonsurgical and surgical strategies, depending on the severity of the condition. nonsurgical treatments involve enhanced oral hygiene, scaling and root planing (SRP), and adjunctive antibiotics. In more advanced cases with persistent deep periodontal pockets and substantial alveolar bone loss, surgical procedures like resective osseous surgery are required (Graziani et al. 2017; Kwon et al. 2021; Łasica et al. 2024).

The pathogenesis of periodontitis is a complex and multifactorial process involving the transition from periodontal health to the chronic stages of periodontitis. Multiple stressors and risk factors, such as microbial biofilm, genetics, smoking, and psychological stress, determine this transition. These factors could induce microbial dysbiosis and dysregulated host immune response that destroys periodontal tissues. Among all, bacterial biofilm is the main etiological factor in periodontitis's initial manifestation (G. Hajishengallis et al. 2020). The first stage of periodontitis development involves the outgrowth of commensal microbiota in response to the nonspecific buildup of dental biofilm in the gingival area, resulting in inflammation and gingivitis. Upon activation of host immune response, polymorphonuclear neutrophils (PMNs) are the first responders, migrating from the bloodstream into the gingival crevice. However, PMNs often struggle to eliminate dysbiotic microbial communities, allowing pathogens to invade deeper connective tissues and triggering the involvement of additional immune cells such as mononuclear phagocytes (MNPs), antigen‐presenting cells (APCs), and T lymphocytes (G. Hajishengallis 2014; Silva et al. 2015). These cells release pro‐inflammatory cytokines, such as TNF‐α, IL‐1β, and IL‐6, heightening the inflammatory response and recruiting additional immune cells that result in tissue damage. In addition, these immune responses activate the adaptive immune system, where effector T cells, particularly Th1 and Th17 subsets, exacerbate inflammation and tissue damage via pathological bone resorption primarily through the receptor activator of nuclear factor κB ligand (RANKL)‐dependent pathway (Kinane et al. 2024; Silva et al. 2015). As the RANKL expression is upregulated, the maturation of osteoclast precursors will be promoted and drive alveolar bone loss (G. Hajishengallis 2014; Pan et al. 2019). In short, the initial host inflammatory response elicited during gingivitis creates an environment that favours the growth of specific pathobionts that thrive in subgingival biofilm, further aggravating dysbiosis and microbial shift and facilitating the emergence of polymicrobial diversity (Van Dyke et al. 2020). The pathobionts, especially *Treponema denticola, Tannerella forsythia, Porphyromonas gingivalis*, and *Actinobacillus actinomycetemcomitans*, can evade host immune mechanisms by generating virulence factors, resulting in a dysregulated immune response. Persistent inflammation will lead to late‐stage periodontitis, which is characterized by the emergence of polymicrobial infection (dominated by anaerobic species) and the decrease in polymicrobial diversity, resulting in uncontrollable inflammation that advances to tissue destruction (Abdulkareem et al. 2023).

Periodontal tissues of healthy individuals are relatively balanced and dominated by a diverse range of planktonic commensal Gram‐positive bacteria species (e.g., *Streptococci*, *Corynebacteria*, *Rothia*, and *Actinomyces* species spp.) (Abdulkareem et al. 2023). They work synergistically under an eubiotic state in the host without eliciting immune responses. On the contrary, periodontitis developed over a broadened timeframe involves the microbial shift of symbiotic Gram‐positive bacteria to Gram‐negative pathogenic bacteria in the subgingival region. A triad of keystone periodontal pathogens in dental plaque biofilm, the so‐called “red complex” comprising of the anaerobic *Porphyromonas gingivalis, Treponema denticola*, and *Tannerella forsythia* has traditionally been proven as the primary etiologic factor to periodontitis with increasing abundance as severity increases (Sedghi et al. 2021). Over the years, bacteria species that cause the manifestation of periodontitis have expanded beyond the red complex, such as *Bacteroides forsythus, Prevotella intermedia, Actinobacillus actinomycetemcomitans, Campylobacter rectus*, and *Fusobacterium nucleatum* (Van Winkelhoff et al. 2002). Although many discrepancies exist between studies, a shift in relative proportions of the four most abundant phyla, including the decreased abundance of Actinobacteria and Proteobacteria and increased abundance of Bacteroidetes and Firmicutes, remains a major defining trait (G. Hajishengallis and Lamont 2012).

#### 4.2.2 Dental Caries (Tooth Decay)

Dental caries, also known as cavities or tooth decay, is a multifactorial infectious oral disease defined as the demineralization and destruction of organic hard tissue of the tooth supragingival region (enamel and dentin) through metabolization of fermentable carbohydrates by cariogenic bacteria owing to microbiome dysbiosis (Ribeiro and Paster 2023). Dental caries is cited by the World Health Organization as a significant public health threat (60%–90% of school children and all adults) (Ndagire et al. 2020). Roughly 2.3 billion adults and 530 million children experienced cavities in the permanent teeth and temporary teeth, respectively (X. Chen et al. 2020). The symptoms of dental caries are facial swelling, bad breath, and toothache. Dental caries can be managed via different methods depending on the location of the lesions within the dental tissues and affected surfaces. Dental sealants are often used as a preventive measure for non‐cavitated pit and fissure caries, although it is only a temporary solution (Cheng et al. 2022; Warreth 2023). For cavitated lesions that do not involve the dental pulp, non‐restorative treatment using silver diamine fluoride (SDF) can be applied; however, this approach carries the risk of permanently black‐staining the treated carious area (Horst 2018). For deep caries reaching the inner pulpal third or quarter of the dentin, restorative materials such as glass‐ionomer cement (GIC) are recommended; however, this could cause possible pulpal irritation (Cheng et al. 2022; Warreth 2023). Although preventable, dental caries can lead to systemic consequences such as swelling that requires emergency hospitalizations and dental extractions if left untreated (Zhan 2018).

Several behavioral, environmental, and sociodemographic risk factors play a significant role in elevating the risk of caries, for instance, oral hygiene practices, high carbohydrate intake (diet), poor education level, low socioeconomic status, lack of fluoride exposure, genetic predisposition, saliva, and antibiotic utilization. Snacking habits between meals and frequent intake or sipping of sugary foods and beverages provide a constant substrate source for acidogenic bacteria and a constantly low pH environment conducive to caries development (Andrysiak‐Karmińska et al. 2022). Besides that, people from low socioeconomic status are often associated with higher caries prevalence as compared to high‐income cohort due to limited access to dental care and education on oral health. Poor oral hygiene practices include improper brushing and flossing, facilitating plaque accumulation on the surface of the teeth, which harbours cariogenic bacteria and increases the risk of caries. Moreover, inadequate exposure to fluoride is an environmental risk factor that increases the teeth's vulnerability to acid attack and microbial dysbiosis (Anil and Anand 2017).

Among the aforementioned, dysbiosis is a significant cause of caries development when the homeostasis of commensal microbiota is disrupted, or their respective functional composition and metabolic activities are affected. Dental caries is mainly attributed to four etiological factors involving a complex interaction between cariogenic microorganisms (factor one) and dietary fermentable carbohydrates (factor two) on susceptible host (factor three) tooth surfaces over time (factor four). Mutans streptococci (MS) is the main cariogenic microorganism genus responsible for the development of dental caries, particularly *Streptococcus mutans* (*S. mutans*) and *Streptococcus sobrinus* (*S. sobrinus*) species. The *S. mutans* (serotypes c, e, f, and k) and *S. sobrinus* (serotypes d and g) are frequently found in human caries lesions (E. Hajishengallis et al. 2017). *Lactobacilli* are also associated with dental caries by playing a specific role in the progression of caries lesions, whereas *S. mutans* is associated with caries initiation by biofilm formation. MS and *Lactobacilli* are acidogenic and capable of producing organic lactic acid by‐products from carbohydrates' fermentative metabolism (Valm 2019). Lactate production reduces pH within biofilm plaque below the critical level of pH 7. The acidic pH environment in biofilm promotes teeth demineralization by dissolving the calcium and phosphate ions of teeth's hard tissues (enamel and dentin) (Andreadis et al. 2015). Moreover, these caries‐associated bacteria can form a biofilm, contributing to bacterial pathogenicity and aggravating the progression of dental caries. Apart from MS, molecular sequencing studies have also demonstrated that other cariogenic bacteria such as *Bifidobacterium* spp., *Scardovia* spp., and *Actinomyces gerencseriae*, along with the fungus *Candida albicans* were also correlated to distinct stages of caries progression in vivo (Aas et al. 2008; Y. Zhu et al. 2023). *Actinomyces gerencseriae* is abundant during the initiation stage, while *Bifidobacterium* spp. was predominant with deep caries lesions (Aas et al. 2008). All these acidogenic and aciduric microorganisms play a crucial role in the pathogenesis of dental caries by shifting supragingival community composition to dysbiosis in the oral cavity (Anil and Anand 2017). Additionally, the outgrowth of acidogenic microorganisms outcompetes *Streptococcus sanguinis* (*S. sanguinis*), a commensal oral bacterium, reducing the abundance of *S. sanguinis* (B. Zhu et al. 2018). However, the antagonistic effects of *S. sanguinis* against *S. mutans* suggest a promising avenue for rebiosis‐based approaches.

## 5 Therapeutic Prospects and Limitations Against Dysbiosis

Inevitably, the lack of a gold standard for diagnosing microbial dysbiosis has led to a scarcity of therapeutic strategies to address the said dysbiosis. Typically, antibiotic treatment has been the preferred choice for dysbiosis such as *Helicobacter Pylori* infection, which ultimately shifts microbiota colonization and reduces the diversity of microbiota species in addition to conferring antibiotic resistance (Alagiakrishnan et al. 2024). Besides, the complete removal of harmful pathogens is impractical as it disrupts the overall microbiome's balance and potentially develops resistance, leading to detrimental consequences. Despite the numerous factors discussed in the context, such as diet, age, lifestyle, and medications, which have considerable potential for dysbiosis treatment, little has been delved into microbiota balance as a potential therapeutic option. As such, this prompts us to highlight the re‐establishment of the commensal microbiota as a mode to remedy dysbiosis, namely rebiosis. Rebiosis entails probiotics, prebiotics, synbiotics, microbiota transplantation, and evolving phage therapy.

Probiotics and prebiotics are common and widely known therapeutics in which probiotics involve the administration of non‐pathogenic live microorganisms. In contrast, prebiotics are non‐digested food products that enhance the growth of commensal microorganisms. Both probiotics and prebiotics aim to restore microbiota homeostasis and confer health benefits to the host (Alagiakrishnan et al. 2024). Given the broad implementation of pre‐ and probiotics, the combination of both was envisioned by many, typically referred to as the synbiotics. Two approaches were presented concerning the synbiotics, namely the complementary and synergistic synbiotics, where the former have both probiotics and prebiotics to confer health benefits to the host and microorganisms independently, and the latter focuses on the coadministration of prebiotics substrate that is selectively utilised by probiotics microorganism to achieve the synergistic effect (Swanson et al. 2020). Due to the adverse impact of the unspecific introduction of commensal bacteria that might lead to disruption of eubiosis, next‐generation (NG) biotics emerged to develop disease‐specific biotics. This is by far achievable through the evolution of the current understanding of multi‐omics approaches such as bioinformatics, metagenomics, and metabolomics of host and microbiota composition (Chang et al. 2019). Some examples of pro‐, pre‐ and synbiotics used for rebiosis to improve various dysbiosis‐related diseases were tabulated in Table 2. While the use of probiotics and prebiotics has proven beneficial, such as the improved gut microbiome community in modulating diabetes (Koutnikova et al. 2019; Zhao et al. 2018), it is a dual‐edge sword where the former may not be suitable for critically ill patients that would otherwise increase opportunistic infection, and the latter brings concerns, especially in the increase of mentioned fermentable colonic bacteria, such as *Ruminococcus* in IBS, aggravating abdominal pain and gas bloating (Didari et al. 2014). Apart from the lack of clinical data, safety‐related data, and limited trials with concrete evidence, synbiotics are by far still at the very infant stage to harness the possible summation of health benefits from pre‐ and probiotics (Swanson et al. 2020).

**Table 2:** Examples of biotics with their beneficial roles in treating various dysbiosis‐related diseases.

Biotics	Outcomes	References
**Probiotics**		
Non‐pathogenic lactic acid bacteria (LAB): *Lactococcus lactis* and *Lactobacillus casei*	Oral delivery of engineered food‐grade LAB strains with human Elafin gene, which was found to be diminished in IBD patients, to mice, resulting in restored colon physiological functions and gut homeostasis, reduced acute and chronic gut inflammation, suggesting a useful treatment for IBD.	Motta et al. (2012)
*Lactobacillus plantarum* CETC 7484 and CETC 7485; *Pediococcus acidilactici* CECT 7483	Oral administration of probiotics together with vitamin D in IBS patients for 42 days resulted in the relief of IBS symptoms, improved life quality, anxiety, depression and gut‐related anxiety.	Jouët et al. (2024)
*Lactobacillus rhamnosus*	Oral administration of 350 mg probiotics resulted in a significant reduction of AD severity over a period of 8 weeks among children aged between 4 and 48 months (Y.‐J. Wu et al. 2017). Oral administration of probiotic in adults with mean age of 33.7 ± 3.3 years for a 12‐week period, resulting in a consequential improvement in the back AV lesions (Fabbrocini et al. 2016).	Fabbrocini et al. (2016), Y.‐J. Wu et al. (2017)
*Lactobacillus salivarius* LS97, *Lactobacillus paracasei* LC86, and *Lactobacillus acidophilus* LA85	Oral administration of probiotics resulted in a reduction of pathogenic bacteria (*Fusobacterium* and *Porphyromonas*) without causing substantial alterations to the salivary and dental plaque microbiota composition, providing a therapy in chronic periodontitis. (Trial registration: Chinese Clinical Trial Registry (ChiCTR) (https://www.chictr.org.cn) under the registration number ChiCTR2300074108.)	L. Wang et al. (2024)
Heat‐inactivated *Bifidobacterium animalis* BB12	In vitro analysis resulted in the reduction of cariogenicity of *Streptococcus mutans*, providing a therapeutic potential for dental caries.	Schwendicke et al. (2014)
**Prebiotics**		
Inulin	Rats supplemented with 7 g/L inulin fiber demonstrated increased abundances of Firmicutes and Actinobacteria but decreased abundance of Proteobacteria, alleviated burn‐induced muscle atrophy and regulated gut microbiota dysbiosis.	(Gao et al. (2024)
Precision prebiotic oligosaccharide mixture (Fructooligosaccharides, xylooligosaccharides, and galacto‐oligosaccharides)	Oral administration of prebiotics by psoriasis patients for the last 8 weeks of a 12‐week study demonstrated improved gut microbiota with a shift towards an anti‐inflammatory profile, alleviating psoriasis.	Buhaș et al. (2023)
Fermented lingonberry juice (FLJ)	In pilot studies, FLJ promoted the growth of oral *Lactobacilli*, restricted the growth of opportunistic oral pathogens (*Candida* and *S. mutants*), suggesting its potential to alleviate periodontitis and IBD.	Pärnänen et al. (2019, 2024)
Polysaccharides from fungi *Trametes versicolor*	Oral administration of the prebiotics to the mice demonstrated significant amelioration of lipid accumulation and steatosis in hepatocytes and restored gut homeostasis, providing a potential to tackle hyperlipidaemia‐associated intestinal flora disorders.	Bai et al. (2024)
**Synbiotics**		
*Lactobacillus rhamnosus* CGMCC1.3724 with inulin	Oral administration of synbiotics twice daily achieves sustaining weight loss in obese women. (ClinicalTrials.gov ID: NCT01106924.)	Sanchez et al. (2014)
*Lactobacillus casei, Lactobacillus rhamnosus, Streptococcus thermophilus, Bifidobacterium breve, Lactobacillus acidophilus, Bifidobacterium longum, Lactobacillus bulgaricus*, and fructo‐oligosaccharide (FOS)	Oral administration of probiotics and 250 mg of FOS prebiotics improved fasting blood glucose (FBG) and insulin resistance with significant increase in serum high‐density lipoprotein (HDL) in insulin resistance patients (Eslamparast et al. 2014). (ClinicalTrials.gov ID: NCT02008838.) Oral administration probiotics and FOS prebiotic for 7 weeks has led to a significant reduction of alanine aminotransferase (ALT), aspartate aminotransferase (AST), γ‐glutamyltransferase (GCT), FBG and inflammatory TNF‐α, NF‐κB p65, and high‐sensitivity C‐reactive protein (hs‐CRP) in patients with nonalcoholic fatty liver disease (NAFLD) (Eslamparast et al. 2014). (ClinicalTrials.gov ID: NCT01791959.)	Eslamparast et al. (2014), Eslamparast et al. (2014)
*Lactobacillus sporogenes*, and inulin	Consumption of synbiotic bread containing 1 × 10^8^ CFU *Lactobacillus sporogenes* with 0.07 g/1 g inulin prebiotic for 8 weeks, three times a day, has led to a significant decrease in serum triglyceride (TAG), very low‐density lipoprotein‐cholesterol (VLDL‐C), total cholesterol/high‐density lipoprotein‐cholesterol (TC/HDL‐C) and a substantial increase in serum HDL compared to control and probiotic bread in type 2 diabetes patients. (Trial registry code: http://www.irct.ir IRCT201311215623N13.)	Shakeri et al. (2014)

Abbreviations: AD, atopic dermatitis; AV, acne vulgaris; IBD, inflammatory bowel disease; IBS, irritable bowel syndrome.

Microbiota transplantation (MT) is revisited to directly modulate the microbiome, building on the concept of faecal microbiota transplantation (FMT) introduced in 1958 (Eiseman et al. 1958). In FMT, faecal matter from a healthy donor is transferred into the recipient's intestinal tract for rebiosis and treating various diseases (Gupta et al. 2016). FMT was first employed in modern medicine when Dr. Eiseman successfully treated patients with pseudomembranous enterocolitis with this technique (Eiseman et al. 1958). In 2013, FMT was well‐known and became a landmark in treating recurrent *Clostridioides difficile* infection (rCDI) with a significantly high cure rate of 94% compared to the conventional antibiotics treatment (31% cure rate) (Van Nood et al. 2013). The patients treated with FMT showed increased faecal bacterial diversity, particularly the increased *Bacteroidetes* sp. and clostridium clusters IV and XIVa, and decreased *Proteobacteria* sp. (Van Nood et al. 2013). FMT has also shown significant beneficial effects in treating various diseases and complications such as IBD, IBS, hepatic encephalopathy, and diabetes (De Groot et al. 2021; J. Li et al. 2022; Singh et al. 2024). To date, the FMT technique has minimal to no serious adverse effects. However, the broad variation of microbial consortia among individuals has led to further exploration, primarily focusing on the MT spectrum beyond FMT with defined microbial consortia cultured for other diseases where FMT is inapplicable. For instance, the first human clinical trial (NCT03018275) topical (skin) MT (SMT) recruiting the commensal bacteria *Roseomonas mucosa* isolated from healthy volunteers and prescreened in vitro and in vivo models has demonstrated decreased *S. aureus* burden and significant alleviation of AD symptoms (Myles et al. 2018). Oral MT (OMT) has also been proposed and studied in vitro biofilm model consisting of the commensal *Streptococcus sanguinis*, showing a significant reduction of the pathobionts (*Fusobacterium nucleatum* and *Porphyromonas gingivalis*) adhesion, providing a potential cure for periodontitis (Gutt et al. 2018). Nonetheless, the research on OMT is still constrained to animal experiments compared to FMT and SMT (Min et al. 2023). Despite MT's promising therapeutics potential, some limitations remain unresolved, such as the substantial interindividual microbiome variability, the difficulty in identifying of the causal microbiome responsible for the diseases due to the broad human microbiome comprising of bacteria, archaea, fungi and viruses, the uncertainty of the stability and sustainability of the transplanted microbiome under selective environmental pressures, to name a few (Bostanghadiri et al. 2024; Junca et al. 2022). Notably, the working mechanisms of MT, safety, administration routes, and dosage require further exploration.

The evolving phage therapy has garnered renewed interest due to the emergence of antibiotic resistance, making therapeutics increasingly challenging. To simplify, bacteriophage (phage) is a virus associated with a target‐specific nature that can be tailored to target pathogenic bacteria without harming the beneficial microorganisms. Bacteriophage also possesses bactericidal activity and proliferates in a localised pattern to kill targeted bacteria precisely via a lytic or lysogenic cycle, preventing bacterial resistance (Natarelli et al. 2023). Phage therapy has been widely applied in regulating gut microbiome, which is stipulated to indirectly modulate skin and oral microbiome through the skin‐gut and gut‐oral axis. Being a virus, phage has rapid replication, low toxicity, high diversity, stability, and adaptability in various environments, making phage therapy a favoured therapeutic option (Y. Li et al. 2024). Pathobiont‐targeted phage therapy's specificity, safety, and efficacy were evaluated with positive outcomes in alleviating IBD (Federici et al. 2022; Titécat et al. 2022). The phage therapy on IBD is currently under active research, in which a few conducted clinical and preclinical trials were summarised by Fujiki and Schnabl (2023) (Fujiki and Schnabl 2023). The capability of precise editing of microbiota by phage expands the research on skin and oral dysbiosis. A few studies have employed phage therapy targeting pathogenic *S. aureus* to alleviate AD (Geng et al. 2024; J. Totté et al. 2017; J. E. E. Totté et al. 2017). In terms of treating periodontitis, phages of *Enterococcus faecalis* have been isolated to tackle Gram‐positive pathogens, revealed effectiveness in preventing root canal infection, and exhibited substantial host specificity and lytic ability (Khalifa et al. 2015; Xiang et al. 2020). A study also showed that a newly isolated SMHBZ8 lytic bacteriophage with a unique lysis cassette inhibited *S. mutans* biofilms, indicating a potential for phage therapy against dental caries (Ben‐Zaken et al. 2021). Given that phage can lyse or promote biofilm formation, the dual‐sided effects must be accounted for in clinical trials. Extensive research is required to understand the host‐phage interaction and their respective induced immune response to ensure the safety of phage therapy as a therapeutic option.

Having to detail the pros and cons of the abovementioned therapeutics (Table 3), the efforts of developing an “ideal microbiome” which focuses on microbial–microbial and microbial–host interaction are far from established, requiring extensive research for a more personalised treatment regimen against dysbiosis. However, with the supplement of artificial intelligence (AI), we can now predict host‐microbiota responses more accurately. For instance, H. Wu et al. (2025) employed machine learning (ML) algorithms to identify *Bifidobacterium pseudocatenulatum* as a potential therapeutic target for managing diet‐induced obesity. In a greater context, ML approaches have demonstrated significant potential in microbiome research by enabling the prediction of host‐probiotic interactions and personalised microbial therapy for the hosts and identification of microbial biomarkers for early diagnosis (Charizani et al. 2024; P. Li et al. 2022; Marcos‐Zambrano et al. 2021). Apart from that, the application of synthetic microbial communities (SynComs) has gained significant momentum as a promising strategy to eliminate pathogenic bacteria and harmful metabolites while promoting the restoration of a healthy microbiome. One notable advancement in this area is the SER‐109, an oral microbiome therapeutic developed by Seres Therapeutics, which has received FDA approval for the treatment of recurrent *Clostridium difficile* (*C. difficile*) infection (rCDI) (Feuerstadt et al. 2022). SER‐109 inhibits *C. difficile* spore germination and bacterial proliferation through targeted microbiome restoration. In a pivotal Phase 3 clinical trial, SER‐109 demonstrated remarkable efficacy, with a recurrence rate of only 12% compared to 40% in the placebo group. Moreover, the safety profile of SER‐109 was comparable to that of the placebo, underscoring its clinical viability (Feuerstadt et al. 2022). It cannot be overstated that the novelty of interventions against dysbiosis must be justified by developing efficient, personalised, and integrative therapy regimens that align with and leverage advancements in science and technology to enhance patient quality of life and meet public health needs. The lack of clinical data, safety, administration, dosage‐related data, and limited trials with concrete evidence add difficulty for mass applications and should be addressed.

**Table 3:** The therapeutic comparisons between biotics, microbial transplantation and phage therapy.

Therapeutics	Pros	Cons	Potential adverse reactions	Clinical preferences	References
Probiotics	Strain‐specific: targeted treatment Generally safe and easy to administer	Strain‐specific: limited generalizability, and the required strains may not be commercially available or approved Poor persistence in the host's microbiome	Bacteraemia or fungemia in immunocompromised patients (rare cases)	Gut dysbiosis (most common), oral cavity and skin	Boyle et al. (2006), Ellis et al. (2019), Guéniche et al. (2010)
Prebiotics	Noninvasive Promote the growth of endogenous beneficial microbes Boost SCFA production, enhancing innate immunity and inhibiting skin and gut pathogens	Nonspecific: may also nourish opportunistic bacteria Some studies reported limited gastrointestinal improvement in IBS patients	Diarrhea (dose‐dependent)	Primarily gut and skin dysbiosis	Al‐Ghazzewi and Tester (2014), Hamer et al. (2008), Wilson et al. (2019)
Synbiotics	Combined benefits of both pro‐ and prebiotics: enhanced survival and persistence of probiotics in the host and improves their functional impact	Efficacy depends on precise matching of composition	Gastrointestinal discomfort	Gut, oral cavity	Bhatia et al. (2025), Skrzydło‐Radomańska et al. (2020)
MT	Highly effective for *C. difficile* infection Can restore full microbial diversity	Invasive procedure Requires strict donor screening	Infections transmission between donor and receiver Immune reactions	Gut (FMT), emerging in skin and oral	FDA (2020), Kelly et al. (2021)
Phage therapy	Highly specific to target bacteria Avoid disruption of commensal microbiota	Narrow host range May develop bacterial resistance	Immune response to phage Regulatory hurdles	Gut, skin, oral cavity	Castillo et al. (2019), Sarker et al. (2016), Singha et al. (2023)

Abbreviations: FMT, faecal microbial transplantation; IBS, Irritable bowel syndrome; MT, microbial transplantation; SCFA, short‐chain fatty acids.

## 6 Conclusion

Bacterial dysbiosis has undoubtedly left a significant impact on the well‐being of millions across the globe. Concerning the rampant growth of bacterial dysbiosis cases, especially in connection to the gut, oral, and skin, there is a call to unravel the roles of microbiota in human diseases and what are the current therapeutic options to combat dysbiosis. Typically, gut dysbiosis contributes to IBS and IBD, attributed to the overgrowth of Enterobacteriaceae such as *E. coli* and loss of *Faecalibacterium*. Skin dysbiosis has significantly contributed to AV and AD, mainly attributed to the pathogenic bacteria *C. acnes* and *S. aureus*, respectively. On the other hand, oral dysbiosis caused by pathobiont red complex and MS led to periodontitis and dental caries, respectively. It is understood that age, diet, lifestyle, medications, and chemical exposures are among the notable factors for dysbiosis; the inevitable lack of strategies for diagnosing microbial dysbiosis due to a lack of gold standards has typically referred to conventional antibiotics for treatment. Still, problems arise from antibiotic use, antibiotic resistance, shifts in microbiota colonization and reduced microbiota diversity. This prompts us to highlight the importance of re‐establishing the commensal microbiota, namely rebiosis, which entails probiotics, prebiotics, synbiotics, microbiota transplantation and the evolving phage therapy, hoping to restore the bacteria balance to remedy dysbiosis. Nevertheless, we should note that the benefits of rebiosis still outweigh the drawbacks, and continuous investment in rebiosis for clinical applications to combat dysbiosis better should be established.

## Author Contributions

**Siau Wui Chin:** conceptualization, visualization, writing – original draft. **Zheng Yao Low:** conceptualization, writing – original draft. **Wei Qi Tan:** conceptualization, writing – original draft. **Adzzie Shazleen Azman:** supervision, writing – review and editing, supervision.

## Ethics Statement

The authors have nothing to report.

## Conflicts of Interest

The authors declare no conflicts of interest.

## Data Availability

Data sharing is not applicable to this article as no datasets were generated or analyzed during the current study.
